# Mechanisms behind variation in the *Clostridium difficile* 16S–23S rRNA intergenic spacer region

**DOI:** 10.1099/jmm.0.020792-0

**Published:** 2010-11

**Authors:** Alexander Indra, Marion Blaschitz, Silvia Kernbichler, Udo Reischl, Guenther Wewalka, Franz Allerberger

**Affiliations:** 1 Institute of Medical Microbiology, Hygiene and Infectious Diseases, University Hospital Salzburg, Mullner Hauptstr. 48, 5020 Salzburg, Austria; 2 Austrian Agency for Health and Food Safety (AGES), Institute for Medical Microbiology and Hygiene, Waehringerstraße 25a, 1090 Vienna, Austria; 3 Institute for Medical Microbiology and Hygiene, University Hospital Regensburg, Franz-Josef-Strauß-Allee 11, 93053 Regensburg, Germany

## Abstract

*Clostridium difficile* infection is an increasing problem in hospitals worldwide, mainly due to the recent emergence of a hypervirulent *C. difficile* strain. *C. difficile* PCR ribotyping, based on size variation of the 16S–23S rRNA intergenic spacer region (16S–23S ISR), is widely used in Europe for molecular epidemiological investigation. The mechanism underlying the 16S–23S ISR size variations in the genome of *C. difficile* is currently not completely understood. To elucidate this mechanism, isolates of six different PCR ribotypes were analysed by cloning and sequencing the 16S–23S ISR. A direct repeat, IB, of 9 bp was detected up to five times in the 16S–23S ISR in all 47 clones investigated. Thirty-five clones displayed differences either by ribotype or by nucleotide sequence. The sequences of the 16S–23S ISR of *C. difficile* showed a uniformly organized structure, composed of a tRNA^Ala^ gene and spacers of 33 and 53 bp separated by the 9 bp direct repeat IB. The results of the study support the hypothesis that this composition is responsible for the length variations seen in the 16S–23S ISR, and indicate that these length variations result from slipped-strand mispairing and intra- and possibly interchromosomal homologous recombination.

## INTRODUCTION

In recent years, *Clostridium difficile* infection has become a major problem in hospital environments worldwide. The occurrence of a hypervirulent strain, PCR ribotype 027, toxinotype III, North American pulsed-field type 1 (NAP1), was first described in Canada (Pépin *et al.*, 2004). In the USA, cases of *C. difficile* type 027 infection have been reported from at least 38 states. By 2008, type 027 had been detected throughout Europe (Kuijper *et al.*, 2008).

Nosocomial transmission and the use of antibiotics are the main drivers of *C. difficile* infection (Pépin *et al.*, 2004). Recently, an assumption of zoonotic transmission was corroborated by repeated demonstrations of an epidemiological connection between PCR ribotype 078 infections emerging in the Netherlands and its occurrence in pigs (Debast *et al.*, 2009; Goorhuis *et al.*, 2008).

Disease due to *C. difficile* is associated with a wide range of clinical manifestations ranging from mild diarrhoea, through moderately severe illness with watery diarrhoea, to life-threatening and sometimes fatal pseudomembranous colitis, which can be accompanied by toxic megacolon or perforation of the bowel.

Several typing methods, such as PFGE, repetitive extragenic palindromic PCR, restriction endonuclease analysis and PCR ribotyping, have been developed for *C. difficile*; however, they are hampered by problems concerning interlaboratory exchangeability, reproducibility and comparability (Killgore *et al.*, 2008). Slipped-strand mispairing, point mutations and insertion of transposon elements are the main mechanisms for the occurrence of distinguishable bacterial subtypes (Brígido *et al.*, 1991; Deurenberg *et al.*, 2007; Levinson & Gutman, 1987). *C. difficile* PCR ribotyping, the most widely used method in Europe, exploits differences in the length of the 16S–23S rRNA intergenic spacer region (16S–23S ISR) (Bidet *et al.*, 1999; Stubbs *et al.*, 1999). The mechanism behind the differences in 16S–23S ISR evolution is currently not completely understood.

It has been reported that *C. difficile* has a highly mobile mosaic genome consisting of mobile genetic elements, mainly as conjugative transposons (Sebaihia *et al.*, 2006). This mosaic nature has been confirmed, but no transposon activity was found as the cause of the 16S–23S ISR size variations in several analysed sequences (Sadeghifard *et al.*, 2006). To elucidate the mechanisms underlying the length variations of 16S–23S ISR sequences in *C. difficile*, we analysed six *C. difficile* isolates of six PCR ribotypes.

## METHODS

### Micro-organisms.

Six isolates comprising six PCR ribotypes were chosen from the strain collection of the Austrian National Reference Center for *C. difficile* in Vienna, Austria. The isolate of ribotype 001 was initially provided by Ed J. Kuijper (Leiden, The Netherlands) and ribotype 176 by one of the authors (U. R.). The remaining isolates, PCR ribotypes AI5 (Austrian isolate 5), 027, 053 and 078, originated from patients in Austria. The pattern of PCR ribotype AI5 was different from that of available reference strains. All isolates were recultivated on cycloserine-cefoxitine agar plates (*C. difficile* agar; bioMérieux) in an anaerobic atmosphere at 37 °C for 48 h.

### Capillary gel electrophoresis-based PCR ribotyping.

Capillary gel electrophoresis-based PCR ribotyping was carried out with primers as described elsewhere (Indra *et al.*, 2008) to confirm the above-mentioned PCR ribotypes. The 16S primer was labelled at the 5′ end with tetrachlorofluorescein. The sample mixture comprised 25 μl HotStar *Taq* Master Mix (Qiagen), 0.3 μl each primer (10 pmol μl^−1^), 20.7 μl water and 1.5 μl DNA. Samples were amplified in a PCR thermocycler by heating at 95 °C for 15 min for initial enzyme activation, followed by 22 cycles of 1 min at 95 °C, 1 min at 57 °C and 1 min at 72 °C, with a final elongation step for 30 min at 72 °C. The PCR fragments were analysed using a 310 Genetic Analyzer (Applied Biosystems) with a 41 cm capillary loaded with a POP4 gel (Applied Biosystems). A 50–625 bp TAMRA ladder (Chimerx) was used as an internal marker for each sample. Injection of samples was carried out at 5 kV over 5 s, with a total running time of 28 min at a run voltage of 15 kV. The size of each peak was determined using Peakscanner software 1.0 (Applied Biosystems).

### Amplification of the 16S–23S ISR.

DNA was extracted from cultures using a MagNA Pure Compact instrument (Roche Diagnostics) according to the manufacturer's recommendations. PCR ribotyping was performed with primers 16S (10 pmol μl^−1^; 5′-GTGCGGCTGGATCACCTCCT-3′) and 23S (10 pmol μl^−1^; 5′-CCCTGCACCCTTAATAACTTGACC′) as described by Bidet *et al.* (1999). Samples were amplified in a PCR thermocycler by heating at 95 °C for 15 min for initial enzyme activation, followed by 35 cycles of 1 min at 95 °C, 1 min at 57 °C and 1 min at 72 °C, with a final elongation step of 5 min for 72 °C. The amplified products were checked by electrophoresis on 1.5 % agarose gels for 4 h at 100 V using a 100–1000 bp ladder (Fermentas) as size standard every ten lanes. The PCR products were purified using a MinElute PCR Purification kit (Qiagen) according to the manufacturer's recommendations.

### Cloning and sequencing of the 16S–23S ISR.

The purified PCR products were ligated into a pDrive cloning vector and transformed into competent cells using a PCR Cloning Plus kit (Qiagen) in accordance with the manufacturer's instructions. Recombinant colonies were picked and grown in thioglycolate broth. Plasmid DNA was extracted from 47 clones showing an insert using a QIAprep Spin Miniprep kit (Qiagen), following the manufacturer's instructions. The 16S–23S ISR was reamplified using the following PCR program: 95 °C for 15 min (hot start), 35 cycles of 94 °C for 30 s, 50 °C for 30 s and 72 °C for 1 min, and 10 min at 72 °C for final elongation. The 50 μl PCR mixture comprised 25 μl Hotstar Master Mix, 1 μl each primer [10 pmol μl^−1^; M13 forward (−40) (5′-GTTTTCCCAGTCACGAC-3′) and M13 reverse (5′-AACAGCTATGACCATG-3′)], 21 μl PCR-grade water and 2 μl extracted plasmid DNA. The PCR products were purified using a MinElute PCR purification kit following the manufacturer's instructions.

Each sequencing reaction contained 4 μl BigDye Terminator v1.1 (Applied Biosystems), 4 μl template DNA, 2 μl M13 forward or M13 reverse primer (10 pmol μl^−1^), 2 μl sequencing buffer and 8 μl water. After an initial activation step at 96 °C, samples underwent 30 cycles of 96 °C for 10 s, 50 °C for 55 s and 60 °C for 4 min. After the sequencing PCR, products were purified using Centri-Sep columns (Princeton Separations) according to the manufacturer's instructions. Sequences were analysed in a 310 Genetic Analyzer, with a 41 cm capillary loaded with a POP6 gel. Injection of samples was carried out at 2 kV over 30 s, with a total running time of 36 min at a run voltage of 15 kV.

### Analysis of sequence data.

Vector sequences were trimmed from all sequences using the program kodon 3.5 (Applied Maths) and, with the same program, sequences were assembled and checked for any conflicts between the overlapping sequences generated with the forward and reverse primers. Sequences were then aligned and stored. The 16S–23S ISR sequence alignment was revised with the GeneDoc multiple sequence alignment program as described elsewhere (Nicholas *et al.*, 1997).

## RESULTS AND DISCUSSION

Molecular typing methods are valuable tools for outbreak investigation and elucidation of evolutionary relatedness (Faria *et al.*, 2008; Ragon *et al.*, 2008). Nevertheless, differing typing methods can yield discordant results (Hanekom *et al.*, 2008). PCR ribotyping is based on fragment length variations in the 16S–23S ISR, although the detailed mechanism underlying the formation of the variations in *C. difficile* is currently unknown. Differences in 16S–23S ISR fragment lengths observed in PCR ribotyping reflect the allelic variants of the rRNA (*rrn*) operon present in the genome of the respective *C. difficile* ribotype (Hanekom *et al.*, 2008).

In the present study, the 16S–23S ISRs of 47 clones were primarily investigated: eight clones from *C. difficile* isolate PCR ribotype 001, 14 clones from 027, three from 053, seven from 078, six from 176 and nine from AI5.

**Fig. 1. f1:**
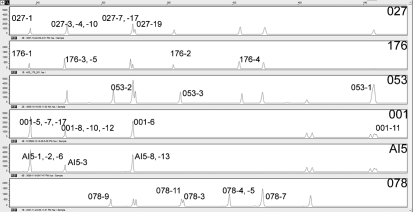
Graphical representation of PCR-ribotyping patterns of all six different PCR ribotypes used in this study carried out by capillary gel electrophoresis-based PCR ribotyping. Cloned and sequenced ISRs are labelled according to the numbers given in Table 3.

**Fig. 2. f2:**
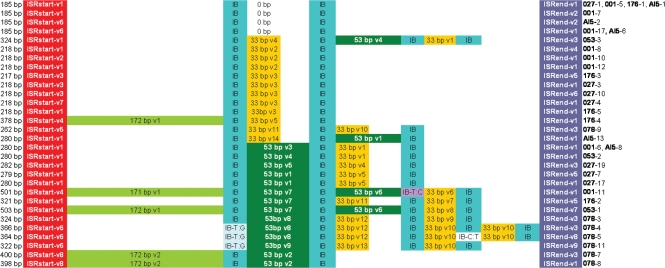
Schematic alignment showing the variants of the 16S–23S ISR of *C. difficile* ribotypes 001, 027, AI5, 176, 053 and 078. ISRstart and ISRend sequences were labelled according to Table 2 and spacer sequences according to Table 1. Direct repeats were designated ‘IB’ and point mutations in the direct repeats were designated ‘IB’ plus the respective mutation.

The 16S–23S ISR sequences ranged in length from 185 to 503 bp. Thirty-five clones showed differences either by PCR ribotype or by nucleotide sequence; 12 clones yielded indistinguishable sequences and these were not included in further investigations. The distribution of sequenced ISR sequences in relation to peaks produced by capillary gel electrophoresis-based PCR ribotyping is shown in Fig. 1. The direct repeat IB with the sequence TTAGCACTT was detected in all cloned 16S–23S ISRs. The direct repeats of a total of five clones displayed point mutations. Clone 4 (366 bp), clone 5 (364 bp) and clone 11 (322 bp) of ribotype 078 showed a T→G (**G**TAGCACTT) point mutation in the first direct repeat, whilst clone 5 showed an additional C→T (TTAG**T**ACTT) point mutation in the fourth direct repeat. Clone 11 (501 bp) of ribotype 001 showed a T→C (**C**TAGCACTT) point mutation in the third direct repeat (Fig. 2).

Sequences between direct repeats (spacer sequences) were either 33 or 53 bp in length (Table 1). Table 2 lists the eight sequences detected at the beginning (before the first repeat or the tRNA^Ala^; ISRstart) and the nine sequences detected at the end of the 16S–23S ISR (after the last repeat; ISRend).

In 30 clones from isolates of ribotypes 001, 027, 053, 176, 078 and AI5, sequence lengths correlated with the number of direct repeats (two to five) present in the 16S–23S ISR: the more direct repeats found, the longer the sequence. However, five clones (176-4, 078-7, 078-8, 001-11 and 053-1) did not correspond to this pattern (italic in Table 3). These five clones had fewer or the same number of repeats than shorter ISR sequences (e.g. clones 078-4 and 001-8; see Table 3) but contained a tRNA^Ala^ gene (73 bp) included in a 172 bp sequence (171 bp in 001-11) located between ISRstart (the beginning of the 16S–23S ISR) and the first direct repeat (Fig. 2).

The occurrence of this gene in the 16S–23S ISR of *C. difficile* has already been described by Sadeghifard *et al.* (2006); moreover, they proposed a mosaic nature for the *C. difficile* 16S–23S ISR, although the issue of the heterogeneity was not addressed. According to the results of our study, the differences in fragment lengths of the *C. difficile* 16S–23S ISR are based on the number of 9 bp direct repeats in the 16S–23S ISR, indicating a highly structured organization of this sequence, in contrast to the findings of Sadeghifard *et al.* (2006).

Our study demonstrated that there are basically two types of 16S–23S ISR in the *C. difficile* genome: 16S–23S ISR sequences with and without the tRNA^Ala^ gene. It remains to be determined whether the gene was originally present in all 16S–23S ISR sequences and subsequently lost during evolution or whether these two types of sequence (with and without tRNA^Ala^ gene) have always existed as two distinct families.

We hypothesize that the structure of the 16S–23S ISR is a product of three mechanisms responsible for variations in *rrn* operons: slipped-strand mispairing, intrachromosomal homologous recombination and possibly interchromosomal recombination.

Slipped-strand mispairing occurs infrequently at positions of direct repeats during bacterial replication (Gürtler, 1999; van Belkum, 1999) and has been described as a mechanism leading to 16S–23S ISR rearrangements in several bacteria of medical importance, such as *Enterococcus faecalis*, *Staphylococcus aureus* and *Escherichia coli* (Gürtler, 1999). The process of slipped-strand mispairing approaches a frequency of 1×10^−4^ per bacterial cell division, resulting in insertion or deletion of short sequence repeats depending on the location of the mispairing (template strand: deletion; nascent strand: insertion; van Belkum, 1999).

**Fig. 3. f3:**
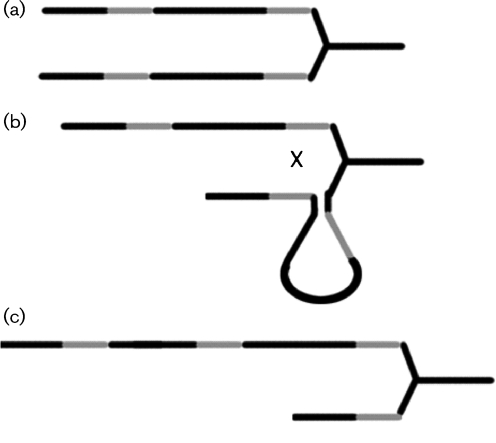
(a) Two identical copies of an *rrn* operon present on the *C. difficile* chromosome. (b) Recombination (shown as an X) at direct repeat positions (shaded line) behind the replication fork. (c) Recombination results in a 16S–23S ISR with a deletion of spacer sequences and another with a duplicated spacer or inverted spacer (depending on the orientation of the two copies to each other).

Intrachromosomal homologous recombination in *rrn* operons has been described previously (Petit, 2005). Our findings showed an inverse spacer arrangement in clone AI5-13 (spacer v1 with 53 bp and spacer v14 with 33 bp) when compared with clones 00-6 and AI5-8 (Fig. 2), typical for intrachromosomal homologous recombination events and shown schematically for *C. difficile* in Fig. 3. This explains why PCR ribotype AI5 was indistinguishable from ribotype 001 by capillary gel electrophoresis-based PCR ribotyping, but showed one band difference in classic agarose gel electrophoresis. This band is a product of incorrect hybridization of different ISRs with similar sequences, as described by Indra *et al.* (2008). The extra band was hypothesized previously to be a result of ‘incorrect’ hybridization of different 16S–23S ISR fragments, as we have now shown with our findings of the inverse spacer arrangement in clone AI5-13, a product of intrachromosomal homologous recombination.

The product of interchromosomal recombination as a chromosome in which the incoming DNA is flanked by direct repeats, also called a ‘pop-in’ recombinant, tends to be unstable, and DNA can ‘pop out’ again unless there is a selective advantage for the newly formed ‘pop-in’ recombinant (Petit, 2005). It is therefore likely that such events seldom lead to stable variations in the 16S–23S ISR. Some strains of *C. difficile* are stated to show interchromosomal recombination (Zaiss *et al.*, 2009); however, we could not find signs of such recombination in the investigated 16S–23S ISRs.

Interestingly, tRNA genes have been described as frequently used sites for bacteriophage DNA insertion (Reiter *et al.*, 1989); thus, the tRNA^Ala^ gene in the 16S–23S ISR of *C. difficile* indicates an insertion site for foreign DNA.

Overall, our study revealed that the 16S–23S ISRs of *C. difficile* show a uniformly organized structure composed of a tRNA^Ala^ gene, spacer sequences of two different lengths and the direct repeat IB of 9 bp. This composition is responsible for the length variations of the 16S–23S ISR used in PCR ribotyping and has evolved as the result of several mechanisms that occur frequently during DNA replication and recombination: slipped-strand mispairing and intra- and possibly interchromosomal homologous recombination.

## Figures and Tables

**Table 1. t1:** Spacer sequence variations found in six *C. difficile* PCR ribotypes PCR ribotypes are in bold. Spacer sequence variants are labelled with consecutive numbers and a prefix of the spacer size in bp and v (variant).

**PCR ribotype-clone**	**Spacer variant**	**Spacer nucleotide sequence**
**001**-6, -8 **AI5**-8 **053**-2, -3	33 bp v1	TAAGCAACGGAATTTATTCGTTGGCGCTGTGCG
**001**-8, -10, -12	33 bp v2	TAAGCAACGGGATTTATTCGTTGGCGCTGTGCG
**027**-3, -4, -10 **176**-3, -5	33 bp v3	TTAGCAACGGGATTTATCCGTTGGCGCCGTGCT
**027**-19 **053**-3	33 bp v4	TAAGCAACGGGATTTATCCGTTGGCGCCGTGCT
**027**-7, -17 **176**-4	33 bp v5	TAAGCAACGGAATTTATTCGTTGGCGCCGTGCT
**001**-11	33 bp v6	TAAGCAACGGAATTTATTTGTTGGCGCCGTGCT
**176**-2	33 bp v7	TAAGCAACGGAATTTATTCGTTGGCGACGTGCT
**053**-1	33 bp v8	TAAGCAACGGAATTTATTCATTGGTGCCGTGCT
**078**-3	33 bp v9	TAAGCAACGGAATTTATTCGTTGGCGCTGTGCT
**078**-4, -5, -9, -11	33 bp v10	TAAGCAACGGAATTTATTCGTTGGCGCTGTGCA
**078**-9 **176**-2	33 bp v11	TAAGCAACGGGATTTATCCGTTGGCGCTGTGCG
**078**-3, -4, -5	33 bp v12	TAAGCAACGTGATTTATCCGTTGGCGCTGTGCA
**078**-11	33 bp v13	TAAGCAACGGGATTTATCCGTTGGCGCTGTGCT
**AI5**-13	33 bp v14	TAAGCAACGGGATTTATCCGTTGGCGACGTGCT
**027**-7, -17 **AI5**-13	53 bp v1	TTAGCAACAGAATAAACTGAACGCATGTGAAGTTTGTTTGTTGGCGCTGTGCG
**078**-7, -8	53 bp v2	TAAGCAACAGAATAAACTGAACACATGTGAAGTTTGTTTGTTGGCGCTGTGCG
**001**-6 **AI5**-8	53 bp v3	TTAGCAACAAAATAAACTGAACGCATGTGAAGTTTGTTTGTTAGCGCTGTGCA
**053**-2, -3	53 bp v4	TTAGCAACAGAATAAACTGAACGCATGTGAAGTTTGTTTGTTGGCGCTGTGCA
**027**-19	53 bp v5	TTAGCAACAGAATAAACTGAACGCATGTGAAGTTTGTTTGTTGGCGTTGTGCG
**001**-11 **053**-1	53 bp v6	TAAGTAACGGAATAATCTGAGTGAATACGAAGGTTGTTCGTTGACGTGGTGCG
**001**-11 **053**-1 **176**-2	53 bp v7	TAAGCAACAGAATAAACTGAACGCATGTGAAGTTTGTTTGTTGGCGCTGTGCG
**078**-3, -4, -5	53 bp v8	TAAGCAACAGAATAAACTGAACGCATGTGAAGTTTGTTTGTTGGCGCTGTGTG
**078**-11	53 bp v9	TAAGCAACAGAATAAACTGAACGCATGTGAAGTTCGTTTGTTGGCGCTGTGTG
**001**-11	171 bp v1	CTTTATATTTGGGGTGTAGCTCAGTTGGGAGAGCACTTGCCTTGCAAGCAAGGGGTCAGGAGTTCGACTCTCCTCATCTCCACCATTTAAGAGTATATTACTTAAATCTTTGATTTACTTAGTAGCCTCTTACAATGCACTTATAGCTTAAATTTATACAAGCTTTGTGTG
**176**-4 **053**-1	172 bp v1	CTTTATATATGGGGGTGTAGCTCAGTTGGGAGAGCACTTGCCTTGCAAGCAAGGGGTCAGGAGTTCGACTCTCCTCATCTCCACCATTTAAGAGTATATTACTTAAATCTTTGATTTACTTAGTAGCCTCTTACAATGCACTTATAGCTTAAATTTATACAAGCTTTGTGCG
**078–**7, -8	172 bp v2	CTTTATATATGGGGGTGTAGCTCAGTTGGGAGAGCACTTGCCTTGCAAGCAAGGGGTCAGGAGTTCGACTCTCCTCATCTCCACCATTTAAGAGTATATTACTTAAATCTTTGATTTACTTAGTAGCCTCTTACAATGCACTTATAGCTTAAATTTATACAGGCTTTGTGCG

**Table 2. t2:** Sequence variations at the beginning and end of the 16S–23S ISR Nucleotide sequences were given numbers with the prefix ‘ISRstart’, ‘ISRend’ and v (variant) for either the beginning or the end sequence of the 16S–23S ISR. PCR ribotypes are in bold.

**PCR ribotype-clone**	**ISR start/end variant**	**Sequence similarity (%) to ISRstart v1 or ISRend v1**	**Start/end of 16S–23S ISR sequence**
**027**-1, -7, -17, -19**001**-5, -6, -7, -8, -12**176**-1, -2, -5**AI5**-1, -8, -13**053**-2, -3**078**-3	ISRstart v1	(100)	AAGGAGAATTACCTACTGTTTAATTTTGAGGGTTCGTTTTTACGAATACTCAAAA
**001**-10	ISRstart v2	98	AAGGAGAATTACCTACTGTTTAATTTTGAGGGTTCGTTTTTACGAGTACTCAAAA
**176**-3 **027**-3, -10	ISRstart v3	98	AAGGAGAATTACCTACTGTTTAATTTTGAGGGTTTGTTTTTACGAATACTCAAAA
**176**-4 **053**-1 **001**-11	ISRstart v4	72	AAGGAGAATTGCCTACTGTTTAATTTTGAAAGTTCTTTACGAA
**078**-9	ISRstart v5	98	AAGGAGAATTACCTACTGTTTAATTTTGAGGGTTCGTTTTTACGAATGCTCAAAA
**AI5**-2, -6 **001**-17**078**-4, -5, -11	ISRstart v6	98	AAGGAGAATTGCCTACTGTTTAATTTTGAGGGTTCGTTTTTACGAATACTCAAAA
**027**-4	ISRstart v7	98	AAGGAGAATCACCTACTGTTTAATTTTGAGGGTTCGTTTTTACGAATACTCAAAA
**078**-7, -8	ISRstart v8	74	AAGGAGAATTACCTACTGTTTAATTTTGAAAGTTTTTTACGAA
**001**-5, -6, -10, -11, -12, -17**027**-1, -3, -4, -17**078**-8**176**-1, -4, -5**AI5**-1, -6, -8, -13**053**-2	ISRend v1	(100)	TGAAAACTGCATATATATTTAGTGATATGACATCTAATTTGTAATATATAAAGCTGATAACTTTTTAAAATTATCGAAGTTGATAGCTTCTAATCTATCAAACCTTTTTAAC
**001**-7 **AI5**-2	ISRend v2	99	TGAAAACTGCATATATACTTAGTGATATGACATCTAATTTGTAATATATAAAGCTGATAACTTTTTAAAATTATCGAAGTTGATAGCTTCTAATCTATCAAACCTTTTTAAC
**027**-19 **053**-3**078**-3, -4, -7, -9	ISRend v3	98	TGAAAACTGCATATATATATTTAGTGATATGACATCTAATTTGTAATATATAAAGCTGATAACTTTTTAAAATTATCGAAGTTGATAGCTTCTAATCTATCAAACCTTTTTAC
**001**-8	ISRend v4	99	TGAAAACTGCATATATATTTAGTGATATGACATCTAATTTGTAATATATAAAGCTGATAACTTTTTAAAATTATCGAAGTTGATAGCTTCTAATCTATCAAACCTTTTTAGC
**027**-7 **176**-2, -3	ISRend v5	96	TGAAAACTGCATATATATTTAGTGATATGACATCTAATTTGTAATATATAAAGCTGATAACTTTTAAAAATTATCAAGTTGATAGACTTTAATCTATCAAACCTTTTTAAC
**027**-10	ISRend v6	99	TGAAAACTGCATATATATTTAGCGATATGACATCTAATTTGTAATATATAAAGCTGATAACTTTTTAAAATTATCGAAGTTGATAGCTTCTAATCTATCAAACCTTTTTAAC
**053**-1	ISRend v7	95	TGAAAACTGCATATATATATTTAGTGATATGACATCTAATTTGTAATATATAAAGCTGATAACTTTTTAAAATTATCAAGTTGATAGACTTTAATCTATCAAACCTTTTTAAC
**078**-5	ISRend v8	95	TGAAAGCTGCATATATATATTTAGTGATATGACATCTAATTTGTAATATAAAGCTGATAACTTTTTAAAATTATCGAAGTTGATAGCTTCTAATCTATCAAACCTTTTTAAC
**078**-11	ISRend v9	96	TGAAAACTGCATATATATATTTAGTGATATGACATCTAATTTGTAATATAAAGCTGATAACTTTTTAAAATTATCGAAGTTGATAGCTTCTAATCTATCAAACCTTTTTAAC

**Table 3. t3:** Sequence lengths and numbers of direct repeats in six *C. difficile* PCR ribotypes PCR ribotypes are in bold. The five clones in which sequence length did not correlate with the number of direct repeats are indicated in italics (see text).

**PCR ribotype-clone**	**Sequence length (bp)**	**Direct repeats (*n*)**
**001**-5, -7, -17**027**-1**176**-1**AI5**-1, -2, -6	185	2
**176**-3	217	2
**001**-8, -10, -12**027**-3, -4, -10**176**-5	218	2
**078**-9	262	3
**027**-7	279	3
**001**-6**027**-17**053**-2**AI5**-8,-13	280	3
**027**-19	282	3
**176**-2	321	4
**078**-11	322	4
**053**-3**078**-3	324	4
**078**-5	364	5
**078**-4	366	5
***176****-4*	*378*	*2*
***078****-8*	*398*	*2*
***078****-7*	*400*	*2*
***001****-11*	*501*	*4*
***053****-1*	*503*	*4*

## Abbreviations

- ISR, intergenic spacer region
